# Deep learning-based image reconstruction and post-processing methods in positron emission tomography for low-dose imaging and resolution enhancement

**DOI:** 10.1007/s00259-022-05746-4

**Published:** 2022-03-21

**Authors:** Cameron Dennis Pain, Gary F. Egan, Zhaolin Chen

**Affiliations:** 1 Monash Biomedical Imaging, Monash University, Melbourne, Australia; 2 Department of Electrical and Computer Systems Engineering, Monash University, Melbourne, Australia; 3 Turner Institute for Brain and Mental Health, Monash University, Melbourne, Australia; 4 Department of Data Science and AI, Monash University, Melbourne, Australia

**Keywords:** PET, Deep learning, Image reconstruction, Low-dose, Denoising, Super resolution, Dynamic PET

## Abstract

Image processing plays a crucial role in maximising diagnostic quality of positron emission tomography (PET) images. Recently, deep learning methods developed across many fields have shown tremendous potential when applied to medical image enhancement, resulting in a rich and rapidly advancing literature surrounding this subject. This review encapsulates methods for integrating deep learning into PET image reconstruction and post-processing for low-dose imaging and resolution enhancement. A brief introduction to conventional image processing techniques in PET is firstly presented. We then review methods which integrate deep learning into the image reconstruction framework as either deep learning-based regularisation or as a fully data-driven mapping from measured signal to images. Deep learning-based post-processing methods for low-dose imaging, temporal resolution enhancement and spatial resolution enhancement are also reviewed. Finally, the challenges associated with applying deep learning to enhance PET images in the clinical setting are discussed and future research directions to address these challenges are presented.

## Introduction

Positron emission tomography (PET) is a highly versatile means of measuring physiological processes in vivo as an investigative tool for scientific discovery and a diagnostic tool for clinical patient care. Advancements in PET stem from a diverse range of fields including physics, radio- and biochemistry, materials science, modelling and data science and many medical disciplines, all of which act to cooperatively improve the efficacy of PET. From a data processing standpoint, advancements in PET imaging come from the development of optimal methods for extracting information from measured signals that are pertinent to clinical diagnosis and quantitative accuracy.

Since the first implementation of PET imaging [1], methods for reconstructing and processing images have been developed to maximise its clinical utility. Conventional image reconstruction and post-processing methods rely on either a physical model of the data acquisition or empirically derived functions in combination with methods of incorporating prior information into the image processing framework. More recently, developments in deep learning have motivated research into methods for incorporating *learned* prior information into medical image processing. Cornerstone works in CT and MRI have shown deep learning can produce state-of-the-art performance in areas such as low-dose imaging [2], super resolution [3], image-to-image translation [4], motion correction [5] and image segmentation [6]. Deep learning methods in PET have subsequently demonstrated exceptional performance in the same tasks. The work will review the literature on deep learning in PET image reconstruction, low-dose to full-dose mapping, temporal resolution improvement and spatial resolution enhancement. These methods optimise the utility of acquired data rather than apply physical corrections such as scatter removal, motion correction or attenuation correction which correct the measured signal as described in recently published reviews [7–9]. 

**Fig. 1:**
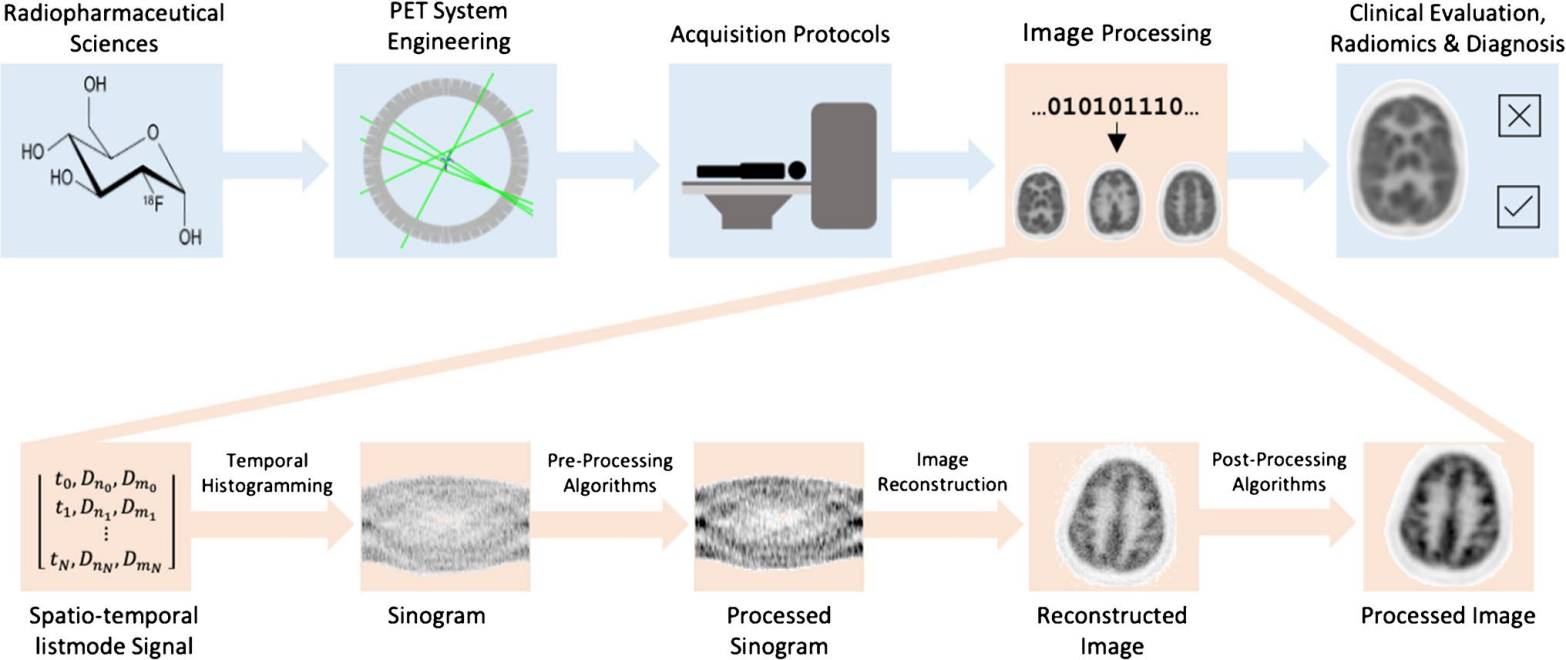
Image processing in the context of PET as a whole. Advancements in various fields contribute holistically to improvements in PET as a modality. This review considers data-driven deep learning-based techniques in the image processing pipeline

Recent reviews of deep learning-based methods in PET have covered both denoising and attenuation correction methods [10], broad scope analysis of diagnostic prediction, segmentation and processing in both PET and CT [11] and a focused introduction and review of deep learning in PET reconstruction [12]. This review aims to encapsulate the current forefront of research in deep learning-based image reconstruction and post-processing for data enhancement as an integral image processing step (Fig. 1). We present a brief introduction on conventional PET image reconstruction and post-processing techniques, followed by an overview of deep learning-based image reconstruction, low-dose to full-dose post-processing, temporal resolution improvement and spatial resolution enhancement. This review summarises the current state-of-the-art artificial intelligence methods in PET image reconstruction and post-processing, and discusses future research directions.

## Search criteria

The works reviewed in the sections “Review of deep learning-based image reconstruction”, “Review of deep learning-based low-dose to full-dose post-processing” and “Review of deep learning-based resolution enhancement” were primarily searched for using the PubMed and Scopus databases with the search terms (“PET” or “PET-MRI” or “PET-CT”) and (“deep learning” or “neural network”) and (“reconstruction” or “low dose” or “low count” or “denoise” or “resolution” or “dynamic” or “temporal”) in the title, abstract or keywords. Additional searches were performed using the same keywords in IEEE journals and nature journals. Lastly, google scholar was used to identify any possible omissions. Several non-peer reviewed archived papers were included due to their relevance to this review. Papers which included the terms “segmentation”, “attenuation correction” and “scatter correction” in the title, abstract or keywords were excluded from the search results. A total of 183 research articles were gathered from the search criteria between January 2016 and December 2021. Finally, works which focused on coregistration, motion correction, hardware level improvements and deep learning-based diagnostic prediction without explicit focus on image enhancement were manually excluded. A total of 80 research articles focused on deep learning-based image processing which fell within the scope of this review were included and 31 works focused on conventional image processing techniques were included.

## Background

In general, PET image reconstruction and enhancement is an ill-posed inverse problem which can be usefully formulated in the following way: Given an observation *y* ∈ Y obtained from the desired latent image *x* ∈ X by some forward model *f*: X → Y derive the estimate $\widehat{x}$ such that:

1 $$\widehat{x}= \underset{x}{\mathrm{argmin}}L\left(y, f\left(x\right)\right)+R(x)$$

where $L$ is a data consistency term which evaluates the difference between observation $y$ and modelled observation $f\left(x\right)$ and $R(x)$ incorporates prior knowledge regarding the nature of $x$. The formulation of Eq. (1) generally describes the steps which constitute the PET image processing pipeline (Fig. 1).

### Maximum *a posteriori* image reconstruction

PET image reconstruction is defined as the method by which an image of the positron annihilation distribution is recovered from the acquired gamma ray coincidence detection signals. Modern state-of-the-art clinical PET image reconstruction is based on a framework first proposed by Shepp and Vardy [13] which estimates the activity distribution that maximises the a posteriori probability of observing the measured PET signal with respect to a model of the PET system. The data acquisition process is modelled as the linear equation:

2 $${s}_{i}=\sum_{j}{a}_{ij}{\lambda }_{j}$$

where the system matrix element ${a}_{ij}$ is the probability of an annihilation event in voxel ${\lambda }_{j}$ being detected in randoms and scatter corrected sinogram voxel ${s}_{i}$. The system matrix is determined by the scanner geometry and incorporates physical models of the system performance [14–16]. Each voxel of the reconstructed annihilation distribution is treated as an independent Poisson distributed random variable based on the nature of radioactive decay. From the conditional probability of measuring sinogram *s* given annihilation distribution *λ*, the negative Poisson log likelihood function defined as:

3 $$-\mathrm{log}P\left(s|\lambda \right)= \sum_{ij}{a}_{ij}{\lambda }_{j}- {s}_{i}\mathrm{log}{a}_{ij}{\lambda }_{j}$$

is derived and used as a convex data consistency term for image reconstruction. Using these formulations of data consistency and a forward model, maximum a posteriori PET image reconstruction in the form of Eq. (1) is defined as:

4 $$\widehat{\lambda }= \underset{\lambda \ge 0}{\mathrm{argmin}}\sum_{ij}{a}_{ij}{\lambda }_{j}-{s}_{i}\mathrm{log}{a}_{ij}{\lambda }_{j}$$

where $\widehat{\lambda }$ is the reconstructed image and no prior constraints on the choices of $\lambda$ are applied.

### Regularisation for image reconstruction

PET images suffer from low signal-to-noise ratio and low spatial resolution relative to anatomical imaging modalities such as MR and CT, due to the intrinsic physical limitations of the PET system design and data acquisition process. The severity of such effects can be mitigated by including prior knowledge of the solution when solving the objective function of Eq. (4). Analytical regularisers are hand-crafted functions used to augment the objective function of Eq. (4)

5 $$\widehat{\lambda }=\underset{\lambda }{\mathrm{argmin}}-\mathrm{log}P\left(s|\lambda \right)+R(\lambda )$$

where the prior model, $R(\lambda )$, introduces constraints on the solution based on prior knowledge of the desired solution, which in the context of PET imaging, commonly involves minimising noise whilst preserving sharp edges [17].

Considerations to make when choosing a prior are the degree to which they improve image quality and the effect they have on the numerical methods used to solve Eq. (5). Priors which constrain pixel values according to a pre-defined probability distribution with a pre-defined expectation value often result in computationally efficient closed form solutions for iteratively solving Eq. (5), however can introduce significant bias into the solution depending on the choice of parameters. Levitan et al. [18] and Lange et al. [19] demonstrated means of constraining voxel values according to Gaussian and Gamma distributions, respectively, and solving Eq. (5) efficiently with faster convergence than unconstrained reconstructions.

A more comprehensive yet computationally expensive choice of $R(\lambda )$ considers spatial dependence of neighbouring voxels such that:

6 $$R\left(\lambda \right)= \sum_{i}^{N}\sum_{j\in {\mathcal{N}}_{i}}{V}_{ij}({\lambda }_{i},{\lambda }_{j})$$

where some potential function ${V}_{ij}({\lambda }_{i},{\lambda }_{j})$ is designed to penalise a voxel value ${\lambda }_{i}$ as a function of surrounding voxel values ${\lambda }_{j}$ in some neighbourhood ${\mathcal{N}}_{i}$ centred on the ${i}^{th}$ voxel. In practice, a number of potential functions have been investigated [20–22], all of which generally aim to penalise large variations in adjacent pixels to minimise noise. Priors which consider intervoxel dependence often require a significantly longer computational time in comparison to spatially independent priors, yet do not require pre-defined choices of mean pixel values.

Synthesis regularisation methods such as kernel-based reconstruction [23, 24] and dictionary matching [25–27] constrain the solution to the reconstruction problem to be synthesised from a predefined basis as follows:

7 $$\widehat{z}=\underset{z}{\mathrm{argmin}}-\mathrm{log}P\left(s|Kz\right)$$

8 $$\widehat{\lambda }=K\widehat{z}$$

where the pre-defined basis vectors defined by the matrix $K$ are derived from prior knowledge of the solution and the optimal combination is determined by $\widehat{z}$. Defining a generally applicable function or dictionary is difficult in practice due to the varying image features from different anatomy, different tracers, and the variance in biological and anatomical characteristics across patients. Simultaneously acquired MR or CT images provide high-resolution anatomical information that is inaccessible by stand-alone PET and can be used to determine the regularisation. Anatomically guided reconstruction can be integrated into analytical priors of the form shown in Eq. (6) where PET voxels can be weighted against simultaneously acquired or coregistered MR images at each iteration of the reconstruction [28–31], to encourage uniformity and edges in the PET image which correspond to those in the MR image. Similarly, simultaneously acquired MR images can be utilised in synthesis regularisation [24, 32] where the matrix $K$ as shown in Eqs. (7) and (8) is derived or dependent on MR information. The quality of the anatomically guided PET reconstruction is therefore determined according to a posteriori knowledge regarding the relationship between the spatial distribution of the PET tracer and the anatomical image contrast.

### Conventional post-processing techniques

A common and often more practical approach to enhancing PET images is to apply additional constraints to images post-reconstruction. Such methods can be applied without any adjustments to existing stages in the image processing pipeline, which are often inaccessible on commercially available software, and provide more a computationally efficient means of iteratively adjusting the parameters of the additional processing step. Post-processing techniques are typically implemented to either control noise or to improve image contrast.

Clinical protocols often control noise by convolving reconstructed images with a simple Gaussian blurring kernel. Although strong noise control is required for applications like dynamic PET or low-dose imaging, heavy Gaussian blurring indiscriminately attenuates both noise and high spatial frequency details in the image. More intricate methods utilise functions of the form in Eq. (6) where spatially dependent relationships between pixels are utilised. Non-local means smoothing which performs a weighted average dependent on both the similarity of coupled pixel values and their distance [33, 34] are often more beneficial in such situations. Block matching methods [35] which define pixel-wise weightings from a set of spatially similar blocks extracted from the same image have also been investigated for PET denoising [36]. Methods more specific to PET imaging such as spatially dependent smoothing with non-negativity constraints have also been developed for applications in low-count PET [37].

The limited spatial resolution of PET imaging systems also leads to significant partial volume effects for high spatial frequency details in PET images resulting in an under-estimation of peak uptake for small features with large contrast and blurring sharp boundaries. Post-processing for reducing partial volume effects is typically implemented using iterative deconvolution methods. Such methods model the point-spread function of the PET imaging system and numerically estimate a high-resolution image consistent with the associated blurring by minimising a function of the form [38–40]:

9 $${\widehat{\lambda }}_{PVC}= \underset{{\lambda }_{\mathit{PVC}}}{\mathrm{argmin}}{\Vert \lambda -h*{\lambda }_{PVC}\Vert }^{2}$$

where $\lambda$ is the initial reconstructed image, ${\lambda }_{PVC}$ is the estimated image with partial volume correction and $h$ is the estimated point-spread function of the imaging system. Iterative deconvolution methods amplify the high-spatial frequencies in the image which includes amplifying noise [41] and can also potentially generate edge artefacts [42]. MR-guided post-processing may simultaneously denoise and perform partial volume correction by leveraging the comparatively low-noise and high-resolution quality of MR images. Reconstructed PET image voxels are weighted based on neighbouring MR image voxels in a pre-defined manner using a function of the form in Eq. (6) [43, 44]. Such methods produce perceptually appealing images with excellent noise control and partial volume correction; however, the quality is strongly dependent on the coregistration between images and the empirically derived way in which the MR contrast relates to PET contrast.

### A deep learning approach to PET image processing

Often it is the case that excellent PET image quality can be produced with long acquisition times and relatively large radiation doses. However, in practice, achieving optimal image quality is infeasible due to the demand for patient throughput and to limit the risks of radiation exposure. Deep learning provides a framework to learn data-driven mappings from low-quality to high-quality images with the aim of using the most logistically beneficial imaging protocols while achieving optimal image quality.

Using a set of high-quality images $Y$ reconstructed with an optimal imaging protocol and a corresponding set of low-quality images $X$ reconstructed with a truncated imaging protocol (faster and lower dose), deep learning-based methods, based on a neural network $N:X\to Y$, aim to generate an estimate of a high-quality output image ${y}_{i}\in Y$ from the corresponding low-quality input ${x}_{i}\in X$ as:

10 $${\widehat{y}}_{i}=N({x}_{i};\Theta )$$

where ${\widehat{y}}_{i}$ is the estimate of ${y}_{i}$ and $\Theta$ is the set of neural network parameters. The parameters of the neural network are iteratively optimised to minimise the loss between the estimated high-quality images and the true high-quality images such that:

11 $$\widehat{\Theta }= \underset{\Theta }{\mathrm{argmin}}\sum_{i}L({y}_{i}, N({x}_{i};\Theta ))$$

where $\widehat{\Theta }$ are the optimal network parameters and *L* is a loss function that quantifies the difference between predicted output and the ground truth in a supervised learning setup. This can further be extended to unsupervised learning setting where the target outputs are unknown, and learning relies on pattern recognition across the set of input data. Information regarding the nature of the high-quality images is encoded into the optimal parameters of the neural network which can be integrated into the maximum a posteriori (MAP) image reconstruction framework or the post-processing pipeline to impose prior knowledge into the final solution.

## Review of deep learning-based image reconstruction

**Fig. 2:**
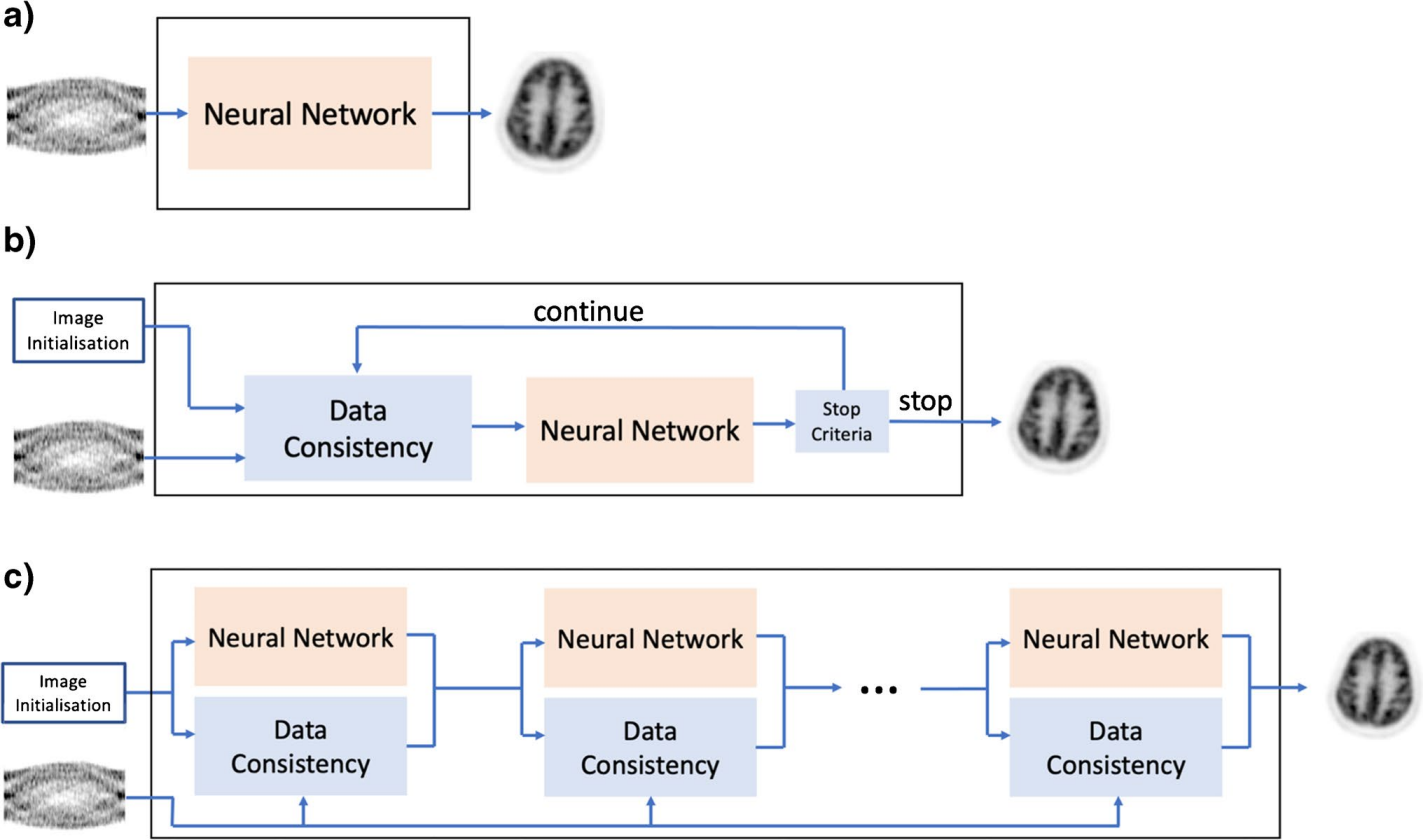
Description of deep learning-based PET image reconstruction methods. **A** End-to-end methods are fully data-driven and do not require an instrument-based system matrix. **B** Regularisation-based methods utilise a neural network in combination with data consistency, retaining the system matrix. **C** Unrolling iterative algorithms into a sequence of processing steps makes it feasible to train iteration-specific regularisation terms

Deep learning-based PET reconstruction methods utilise deep neural networks in mapping raw data to diagnostic images. A neural network can trained to learn a mapping from raw data directly to the desired output image in an end-to-end manner, providing a purely data-driven alternative to conventional image reconstruction methods. Alternatively, existing iterative reconstruction frameworks can be modified to incorporate a neural network as regularisation in combination with data consistency. Figure 2 overviews and compares different deep learning-based image reconstruction methods.

### End-to-end

End-to-end deep learning-based PET reconstruction methods (Fig. 2a) attempt to map sinogram data directly to the corresponding image space data with a neural network:

12 $$\lambda =N(s; {\Theta }_{N})$$

where parameters ${\Theta }_{N}$ of network $N$ are trained to optimally map sinogram $s$ to their corresponding positron annihilation distribution $\lambda$. In contrast to iterative reconstruction techniques which require an accurate model of the forward process, end-to-end deep learning-based approaches can be considered analogous to analytical reconstruction methods such as filtered back projection (FBP) in so far as they attempt to fully characterise the inverse process in a single step, without an explicit model of the forward process. The data-driven nature of such an approach provides a means of overcoming the limitations and potential errors in a well-defined system model, but at the cost of discarding any useful information it provides.

Several works utilise fully convolutional networks for performing the domain transform. For approaches which utilise a fully connected domain transform layer, the network architecture can be generally described as a composition of three modules:

13 $$\lambda =I(D\left(S\left(s;{\Theta }_{S}\right);{\Theta }_{D}\right);{\Theta }_{I})$$

where ${z}_{s}=S(s; {\Theta }_{s})$ is a sinogram processing module, ${z}_{d}=D({z}_{s}; {\Theta }_{d})$ is a learned transform from sinogram space to image space and $\lambda =I({z}_{d}; {\Theta }_{I})$ is an image processing module. Haggstrom et al. [45] presented an end-to-end mapping of PET sinograms to images termed DeepPET using a modified Unet trained on synthetic XCAT digital phantom data. The peak signal-to-noise ratio (PSNR), root mean squared error (RMSE) and structural similarity index (SSIM) outperformed ordered subset expectation maximisation (OSEM) and filtered back projection (FBP) reconstruction for the synthetic evaluation data providing a strong proof of concept. Huang et al. [46] used a fully convolutional network for reconstruction and incorporated a pre-processing neural network for filling crystal spacing gaps in sinogram data. Fully convolutional generative adversarial networks (GAN) were investigated by Liu et al. [47] using a conditional GAN and Hu et al. [48] using a cycle consistent GAN [49] with a VGG19 network [50] trained with clinical data for perceptual loss. Results demonstrated reduced bias and variance relative to FBP and maximum likelihood expectation maximisation (MLEM) reconstruction and the cycleGAN implementation outperformed an instance of the DeepPET architecture in PSNR and SSIM. Kandarpa et al. [51] proposed an architecture consisting of serial models for denoising, image reconstruction and super resolution trained on whole body 18F-FLT images. The authors compared the performance to the DeepPET network and showed improved RMSE and SSIM. Although fully convolutional neural networks are readily available for direct image reconstruction in an end-to-end manner, their overall performance may be limited due to the underlying signal differences between measured sinogram space and reconstructed image space.

Zhu et al. [52] presented a neural network termed AUTOMAP for mapping general sensor space data to image space. The AUTOMAP architecture performs a domain transformation to using three feed forward fully connected layers followed by serial convolutional operations. In the context of PET, fully connected layers define a learned inverse system matrix which can model the non-local relationship between pixels in sinogram and image space. While the authors present only limited examples of application to PET, the concept of learning a fully connected domain transform was subsequently adopted by work specifically aimed at reconstructing PET sinogram data [53–56]. Wang et al. [53] presented a neural network termed FBP-Net which learns a frequency domain filter for sinograms, a fully connected layer for learning a back projection and finally a denoising convolutional neural network (CNN). The network was trained on data derived from a digital phantom augmented with rotations, translations, and scaling. Comparisons were made with DeepPET and a Unet for evaluation, with the FBP-Net being more robust to overfitting and previously unseen anatomy. Other works incorporated unfiltered back projections as a domain transform with no sinogram space filtering [54, 55]. Zhang et al. [54] presented the bpNET which used an unfiltered back projection as a pre-processing step followed by a residual encoder decoder network trained on synthetic data. Xue et al. [55] also use an unfiltered back projection as pre-processing with a cycle consistent GAN network [*cycleGAN]* trained on clinical data. Whiteley et al. [56] presented a network termed Direct-PET which learned an optimal sinogram compression and performed a more efficient domain transform by masking the sinograms and mapping to a patch in image space. Neural network architectures which incorporate either learned or pre-defined domain transforms can better account for the non-local relationship between voxels in sinogram and image domains and may present a more viable alternative to fully convolutional neural networks.

Other works [57, 58] which focused on real-time reconstruction have investigated analytically histogramming listmode data directly to image space,providing a raw data representation with a spatially local relationship to a high-fidelity reconstructed image in an attempt to avoid implementing a data-intensive learned domain transform matrix. Whitely et al. [57] presented a network termed Fast-PET which used a modified Unet to map histogrammed image data to a high-quality reconstructed image for real-time PET image reconstruction. They demonstrated reconstructed images comparable to standard OSEM reconstructions with a 67 × reduction in computational time. Methods similar to those presented in [57, 58] are likely to be of considerable interest in applications in which near real-time image reconstruction is required.

### Regularisation based

Regularisation-based deep learning image reconstruction integrates a neural network into the iterative reconstruction framework to constrain the solution with a combination of data consistency and prior information. Retaining the explicit system model helps strengthen the generalisability of the reconstruction algorithm. Recurrent neural networks (RNN) [59] integrate learned parameters into a recurrence relation to model sequential data. The sequential nature of iterative image reconstruction lends itself well as an application of RNNs which unrolls the iterative algorithm into a set of sequential blocks. Gong et al. [60] incorporated a pre-trained neural network into an iterative reconstruction framework which mapped low-count PET images to full-count PET images where the reconstructed image was constrained to be synthesised from a network output (as shown in Fig. 2b). The regularised image reconstruction is given as:

14 $$\widehat{z}= \underset{z\ge 0}{\mathrm{argmin}}L(s, N\left(z;\Theta \right))$$

15 $$\widehat{\lambda }= N\left(\widehat{z};\Theta \right)$$

where $\widehat{\lambda }$ is the final reconstructed image, $s$ is the sinogram input, $\widehat{z}$ is the optimal network input, $N$ is the pre-trained low-dose to full-dose neural network and $L$ is the Poisson log-likelihood. The constraints of Eqs. (14) and (15) are reformulated as a constrained optimisation problem, written as an augmented Lagrange function, and solved using the alternating direction method of multipliers (ADMM) algorithm. An example of Unets trained with clinical brain, heart and lung data was presented demonstrating better contrast recovery and denoising properties than a Unet for post-processing. Subsequent work extended the framework from [60] to a Patlak parametric reconstruction [61]. The same formulation as [60] was also used for implementing unsupervised image reconstruction [62]. Other works [63, 64] subsequently investigated variations of this method including using a GAN for improved perceptual quality and using PET CT data with a modified Unet with separate encoders and a shared decoder.

**Fig. 3:**
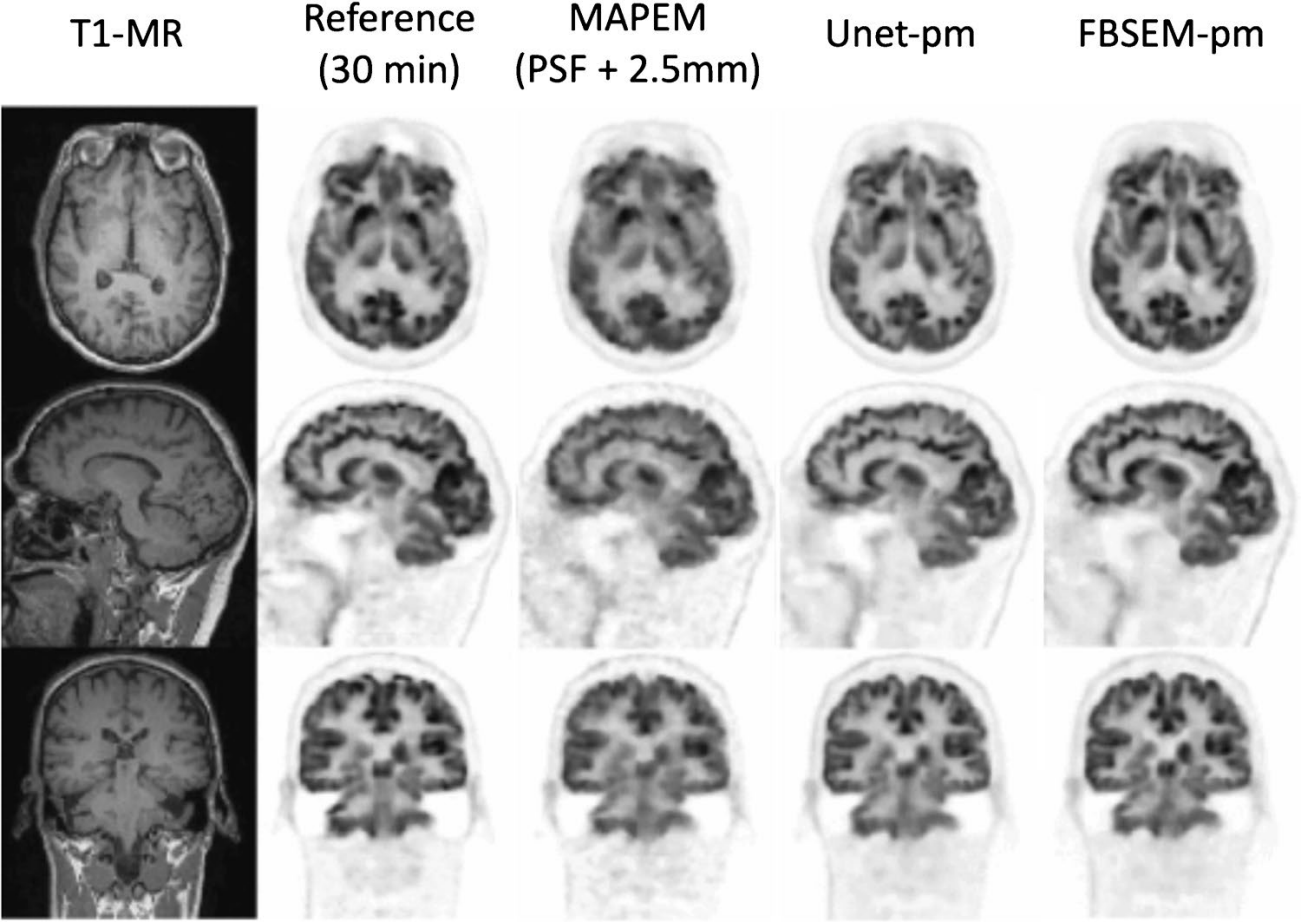
Images from Mehranian et al. comparing reference thirty minute maximum a posteriori expectation maximisation reconstruction with a 2-min reconstruction with FBSEM-Net using PET and MR inputs (FBSEM-pm), standard data consistency-based reconstructions (MAPEM) and deep learning-based post-processing with PET and MR inputs (Unet-pm) (images from [68])

An additive regularisation term which minimises the difference between a neural network synthesised image and the reconstructed image provides a more lenient constraint compared to that used in [60]. Kim et al. [65] utilised a pre-trained neural network in an additive regularisation term with a patch-wise linear mapping of the form:

16 $$R\left({\lambda}^n\right)=\left\Vert \lambda -\left({q}_{\lambda}^n\odot N\left({\lambda}^n;\Theta \right)+{b}_{\lambda}^n\right)\right\Vert$$

where ${\lambda }^{n}$ is the current estimate of the image at iteration $n$, $N$ is a neural network trained with parameters $\Theta$ to map low-dose to full dose images, ⨀ is pixel-wise multiplication and ${q}_{\lambda }^{n}$ and ${b}_{\lambda }^{n}$ are matrices of local linear fitting parameters derived from the input $\lambda$. The linear fit reduces bias by ensuring mean pixel values within small regions are consistent with the original input. Similar work by Wang et al. [66] used a relative difference in the regularisation with no local linear fitting. Lv et al. [67] developed a formulation which integrated two neural networks into the MAP framework: an initial denoising network trained to map low count images to full count images and a subsequent image enhancement network trained to map reconstructions with low iterations to high iterations. Denoising network outputs are combined with inputs using an edge preserving step and enhancement network outputs were combined with inputs using a weighted sum, providing a regularisation like Gong et al. [60] which can be fined tuned with a hyperparameter like the regularisation used by Kim et al. [65]. Mehranian and Reader [68] unrolled a forward–backward splitting expectation maximisation (FBSEM) algorithm into a recurrent neural network (FBSEM-Net) using the same residual block at each iteration. Separate instances were trained with synthetic data and clinical ^18^*F*-FDG brain data reconstructed with MR-guided MAP expectation maximisation as ground truth and compared against conventional reconstruction methods and a 3D Unet as post-processing. FBSEM-Net demonstrated comparable performance in enhancing contrast-to-noise ratio, lesion uptake error and normalised RMSE (NRMSE), when trained on synthetic data. Results from FBSEM-Net trained on clinical data (Fig. 3) demonstrated optimal performance in replicating regional activity measures for a 30-min scan when applied to data from the first 2 min of the acquisition despite that deep learning-based processing biased the biodistribution of tracer after 2 min of acquisition towards that of the full 30-min acquisition. Incorporating deep learning-based regularisation as an additive constraint or in the manner of Eqs. (14) and (15) allows for relatively easy integration with model-based iterative reconstruction methods. By retaining the robust performance of the system model, regularisation methods such as these are likely to be a more feasible option than end-to-end reconstruction for clinical application in the near future.

While RNNs conventionally share parameters across all blocks of the network, training parameters unique to each block provides more flexibility in learning an optimal convergence (Fig. 2c). The first application of an RNN with unique parameters at each block was by Lim et al. [69] who applied a modified version of the block coordinate descent network [70] to low-count PET image reconstruction. Convolutional filters unique to each iteration of the reconstruction were trained using synthetic ^90^*Y* liver images to minimise the *L*_2_ norm between the current iteration estimate and ground truth images, demonstrating better performance than total variation regularisation and non-local means. MAPEM-Net from Gong et al. [71] performs a regularised MAP expectation maximisation image reconstruction using an unrolled ADMM algorithm with a 3D Unet as the regularisation term. The algorithm was trained end-to-end on digital ^18^*F*-FDG PET brain phantoms, producing images with significantly better contrast recovery and noise properties compared to the 3D Unet used for post-processing and standard EM reconstruction with Gaussian blurring. Follow-up work from the original FBSEM-Net [72] implemented iteration-specific convolutional kernels and unrolled more iterations with sequential training. Iteration-specific FBSEM-Net was trained on simulated ^18^*F*-FDG PET brains and compared with deep learning-based post-processing, Tikhonov regularisation, the original FBSEM-Net, and an instance of the block coordinate descent algorithm from Lim et al. [69] and demonstrated superior performance in NRMSE and bias-standard deviation trade off. Iteration-specific parameters demonstrated improved robustness to varying noise inputs and tissue contrasts. Sequentially training parameters of each iteration showed equivalent performance for end-to-end training but with a significant decrease in memory requirements for training networks with many unrolled blocks.

## Review of deep learning-based low-dose to full-dose post-processing

Deep learning-based low-dose to full-dose image post-processing refers to methods which utilise neural networks to synthesise full-dose images from reconstructed low-dose images as:

17 $${\lambda }_{fd}=N({\lambda }_{ld};z,\Theta )$$

where $N$ is the neural network trained with an optimal set of parameters $\Theta$, ${\lambda }_{ld}$ is the low-dose input, ${\lambda }_{fd}$ is the synthesised full dose image and $z$ is a potential additional input which contributes information regarding the nature of ${\lambda }_{fd}$. Deep learning-based post-processing techniques are typically more easily implemented than regularised image reconstruction and offer more flexibility than end-to-end reconstruction, and thus present an efficient means to utilise deep learning in practice. The dose reduction factor achievable varies depending on several factors including the information provided to the network, regularisation of the network loss function and the generality of the evaluation dataset. Table 1 provides a summary of deep learning models, datasets and evaluation metrics for the low-dose to full-dose post-processing applications reviewed in this work.

**Table 1:** Summary of deep learning based low-dose to full-dose post-processing implementations reviewed in this work. Details from each source are: the neural network architecture, dimensions of the input data, additional input information, tracer, anatomical region, activity and acquisition time, the dose or time reduction factor, and the evaluation metrics used to convey performance

	Network architecture	PET input dimensions	Additional input data	Tracers	Anatomy	Activity/Acq, time (MBq, min)	Dose/time reduction factor	Evaluation metrics
[73]	CNN	2D Patch	T1	^18^F-FDG	Brain	(203,12)	4	PSNR, nMSE
[74]	Unet	2.5D	None	^18^F-FDG	Brain	(370,40)	200	SSIM, PSNR, NRMSE
[75]	Residual Unet	2D	T1, T2, FLAIR	^18^F-FBB	Brain	(330, 20)	100	PSNR, RMSE, SSIM, QCS, rSUV, CD
[76]	Unet	3D	CT	^18^F-FDG	Cardiac	(300,10)	10, 100	LVEF, ESV, EDV
[77]	Modified Unet	2.5D	LAVA	^18^F-FDG	Whole body	Site 1: (3 kg^−1^, 3.5 bed^−1^) Site 2: (3 kg^−1^, 4 bed^−1^)	16	PSNR, NRMSE, SSIM, rSUV, CTD
[78]	Unet	3D Patch	None	^18^F-FDG	Brain	(5.18 kg^−1^, 5 bed^−1^)	7.5, 30	SNR, SSIM
[79]	CNN (Dilating convolutional kernels)	2D	None	^18^F-FDG	Brain	(166.5, 10)	10	MAE, PSNR, SSIM, rMAE
[80]	Unet	3D	None	^18^F-FDG	Brain	(205, 20)	20	PSNR, RMSE, SSIM, rSUV, QCS
[81]	FFNN	2D Patch	None	Sim ^82^Rb, ^82^Rb	Cardiac	(N/A, 7)	7, 3.5, 1.5	NMSE, ROI Contrast
[82]	Unet	3D Patch	None	^18^F-FDG	Whole body	(225.3, 10)	6.7, 9.1, 13.3, 17.5, 26.3, 66.7, 125, 250, 500	Lesion SUV, QCS, CTD
[83]	Modified Unet	2D	Sim T1	Sim ^18^F-FDG	Brain	(N/A, N/A)	N/A	MSE, Lesion CR
[84]	CNN	3D	T1	^18^F-FDG	Brain	(N/A, N/A)	10, 100	NRMSE, SUV bias
[85]	cycleGAN	2D Patch	None	^18^F-FDG	Brain	(218.3, 20)	125	PSNR, NRMSE, SSIM, SUV bias
[86]	GAN	2D	None	^18^F-FBB	Brain	(300, 20)	10	PSNR, NRMSE, SSIM, rSUV, QCS, CD
[87]	GAN	3D Patch	None	^18^F-FDG	Whole body	(5.55 kg^−1^, 20)	2	SSIM, PSNR
[88]	cycleGAN	3D Patch	None	^18^F-FDG	Whole body	BMI $\le$ 18.5: (370, 1.5 bed^−1^) $18.5\le$ BMI $\le$ 25: (370, 2 bed^−1^) $25\le$ BMI $\le$ 30: (370, 2.5 bed^−1^) 30 $\le$ BMI: (444, 2.5 bed^−1^)	8	MAE, NRMSE, rPSNR
[89]	cycleGAN	2D Patch	None	^18^F-FDG	Whole Body	(370, 5)	3.3, 10	PSNR, NRMSE SUV bias
[90]	GAN	2D Patch	None	^18^F-FDG	Whole body	(N/A, N/A)	10	PSNR, RMSE, SSIM Lesion SUV
[91]	GAN	2.5D	None	^18^F-FBB	Brain	(330, 20)	100	PSNR, RMSE, SSIM, FBM, EBM, CD
[92]	GAN	3D Patch	None	^18^F-FDG	Brain	(203, 12)	4	PSNR, nMSE, rSUV
[93]	GAN	3D Patch	T1, DT	^18^F-FDG	Brain	(203, 12)	4	PSNR, SSIM, rCR
[94]	GAN	3D Patch	None	^18^F-FDG	Whole body	(5.55 kg^−1^, 20)	5	NRMSE, PSNR, RFSIM, VIF
[95]	CAE, Unet, GAN	2D, 2.5D, 3D	None	^18^F-FDG	Thoracic	(370, 20)	10	PSNR, nMSE, Lesion SUV bias
[96]	Residual Unet	2D	T1, T2, FLAIR	^18^F-FBB	Brain	LD: (8, 30) FD: (334, 20)	42	PSNR, RMSE, SSIM rSUV, QCS, CD
[97]	Residual Unet	2D	T1, T2, FLAIR	^18^F-FBB	Brain	Site 1: (330, 20) Site 2: (283, 20)	Site 1: 100 Site 2: 20	PSNR, RMSE, SSIM rSUV, QCS, CD
[98]	Unet	3D Patch	None	^18^F-FDG, ^18^F-FMISO, ^68^Ga-Dotatate	Whole body	FDG: (340, 20) FMISO: (181, 50) DOTATATE: (130, 21.6)	10	PSNR, NRMSE, Lesion SUV bias
[99]	Residual Unet	2.5D	None	Sim ^18^F-FDG, ^18^F-FDG	Brain	(185, 70)	4	CR
[100]	Unet	2.5D	None	^18^F-FDG	Whole body	Site 1: (481, 3 bed^−1^) Site 2: (400, 3 bed^−1^) Site 3: (429, 3 bed^−1^)	4	QCS, CTD, rSUV
[101]	Residual Unet	3D	None	^18^F-FDG	Whole body	(391, 2.45 bed^−1^)	1.33, 2, 4	CTD, rSUV
[102]	Modified Unet	2.5D	T1, T2	^18^F-FDG	Brain	(230, 30)	180	PSNR, SSIM

*DT* diffusion tensor, *PSNR* peak signal-to-noise ratio, *RMSE* root mean square error, *NRMSE* normalised root mean square error, *MSE* mean square error, *MAE* mean absolute error, *rSUV* regional SUV, *CR* contrast recovery, *SSIM* structural similarity index, *QCS* qualitative clinical score, *CD* clinical diagnosis, *CTD* clinical tumour detectability, *LVEF* left ventricular ejection fraction, *EDV* end diastolic volume, *ESV* end systolic volume, *LAVA* liver acquisition volume acceleration, *RFSIM* Riesz-transform based feature similarity, *VIF* visual information fidelity, *Sim* simulated data

### Single modality PET input data

Deep learning models which require only PET images as an input provide a versatile means to implement low-dose PET post-processing on hybrid or stand-alone PET systems.

The first report that demonstrated orders of magnitude of dose reduction with PET only input data by Xu et al. [74] used a 2.5D Unet with supervised residual learning to map low-dose ^18^*F*-FDG PET brains to full-dose. They achieved a 200 × dose reduction factor that demonstrated superior PSNR, NRMSE and SSIM as compared to non-local means and 3D block matching. Several subsequent papers further developed the application of neural networks for PET only data. Sanaat et al. [80] compared low-dose to full-dose mappings with a 3D Unet in image space and sinogram space and demonstrated that sinogram space processing produced improved results with significantly higher PSNR and significantly lower SUV bias. Spuhler et al. [79] used a CNN formulation with dilation of the convolutional kernels in place of down-sampling operations, with PSNR, SSIM and NRMSE results comparable to a Unet. Wang et al. [81] trained a feed-forward fully connected network with synthetic data to denoise low-dose 4 × 4 × 4 pixel patches and directly applied the network to ^82^*Rb* cardiac PET images. Schaefferkoetter et al. [82] trained a 3D patch-based Unet to map low-dose to full-dose images of patients with small-cell lung cancer and evaluated SUV bias in lesions. Fully supervised methods such as these have demonstrated the ability to reproduce quantitative image metrics consistent with standard-dose PET images from relatively large dose reduction factors; however, implementation requires a comprehensive set of training data to help ensure performance translates generally to a clinical environment.

Unsupervised learning and methods which reduce the required number of high-quality training pairs are of interest in data scarce applications and situations where high-quality data is unattainable. The deep image prior (DIP) formulated by Ulyanov et al. [103] that parameterised a single noisy image with a neural network based on self-supervised training was used by Cui et al. [104] as a post-processing technique for noisy whole body PET images. DIP-based methods produced better contrast-to-noise ratio compared to the deep decoder method from Heckel et al. [105], Gaussian smoothing, non-local means and 4D block matching. Work from Cui et al. [106] extended this concept by implementing the DIP method with a neural network initially trained on population level information, demonstrating better results than a randomly initialised DIP. Furthermore, Yie et al. [78] investigated the quality of supervised learning approaches with noisy target images. Transferred learning across imaging protocols and tracers was investigated by Liu et al. [98] for implementing deep learning-based low-dose processing with limited training data. Transferred learning from ^18^*F*-FDG to ^68^* Ga*-DOTATATE and ^18^*F*-FMISO demonstrated a reduction in training data required to achieve consistent performance with ^68^* Ga*-DOTATATE and ^18^*F*-FMISO networks trained from scratch. Directly applying neural networks trained with FDG images to 18F-Florbetapir images, 18F-FET images and across scanner manufacturers has also demonstrated statistically significant improvements in image quality for dose reduction factors ranging from 2 to 100 [107]. Unsupervised learning methods generalise well across various clinical situations; however, they do not incorporate population level information to the extent of supervised learning which limits their ability to achieve comparable levels of dose reduction. The continued development of learning methods which reduce the required amount of training data and that generalise well to unseen data will be crucial to expanding the clinical impact of deep learning.

**Fig. 4:**
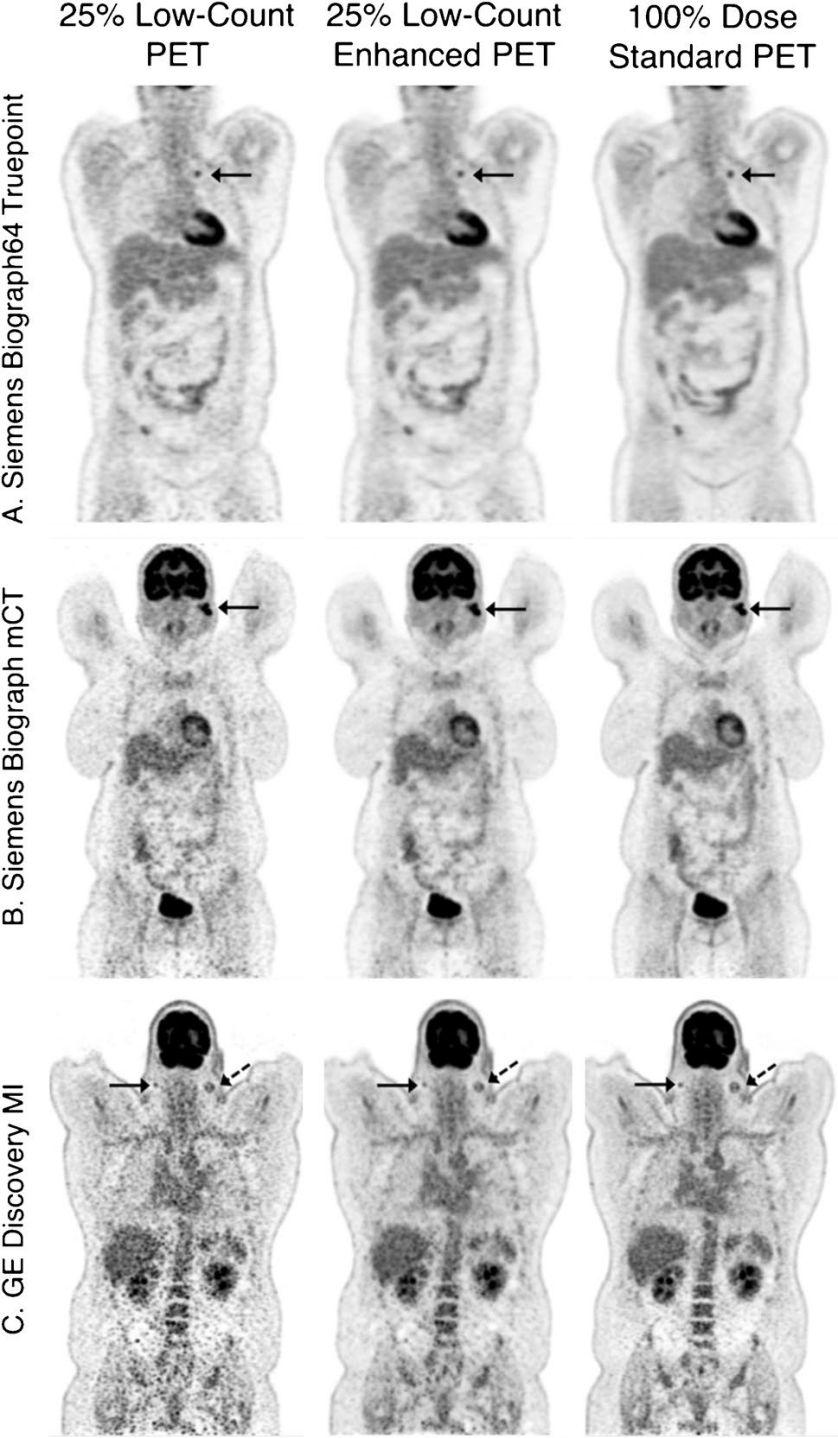
Examples of deep learning-based low-dose to full-dose post-processing using PET only inputs and evaluated across multiple sites using different acquisition protocols and scanners. The neural network used in this case was trained using data sourced independently from the three evaluation sites to provide an unbiased evaluation (Image from [100])

Studies have also investigated the performance of deep learning across sites with varying imaging protocols. Mehranian et al. [101] evaluated the performance of a 3D residual Unet for mapping short duration whole body ^18^*F*-FDG scans to full duration scans using data from six sites. Results demonstrated improved lesion quantitation and detectability with radiation dose reductions of 50%. Chaudhari et al. [100] evaluated the performance of a 2.5D Unet on whole body ^18^*F*-FDG scans collected across three sites with a dose reduction factor of 4 × (Fig. 4). Training data was sourced entirely from outside the three evaluation sites to provide an unbiased evaluation of deep learning techniques across sites. Synthesised full-dose scans demonstrated comparable lesion detectability, qualitative clinical scores and SUV accuracy to standard full dose scans which indicated neural networks may be sufficiently generalisable to realise significant dose reduction across centres without additional fine tuning. Future studies which demonstrate the extent to which deep learning-based methods can generalise across sites will be necessary for identifying the feasibility of clinical implementation beyond those facilities with the means for in-house development. Such studies will also help to guide the development of commercially viable products to help make deep learning-based methods widely available.

### Multi-modality input data

While deep learning has demonstrated feasibility in low-dose post-processing with only PET inputs, the relatively poor image quality intrinsic in low-dose PET limits the information available for learning. Hybrid systems including PET/MR and PET/CT can simultaneously or sequentially acquire MR and CT images, which can be used as additional inputs to the neural network to leverage the high-quality anatomical information inherent in these modalities [108].

**Fig. 5:**
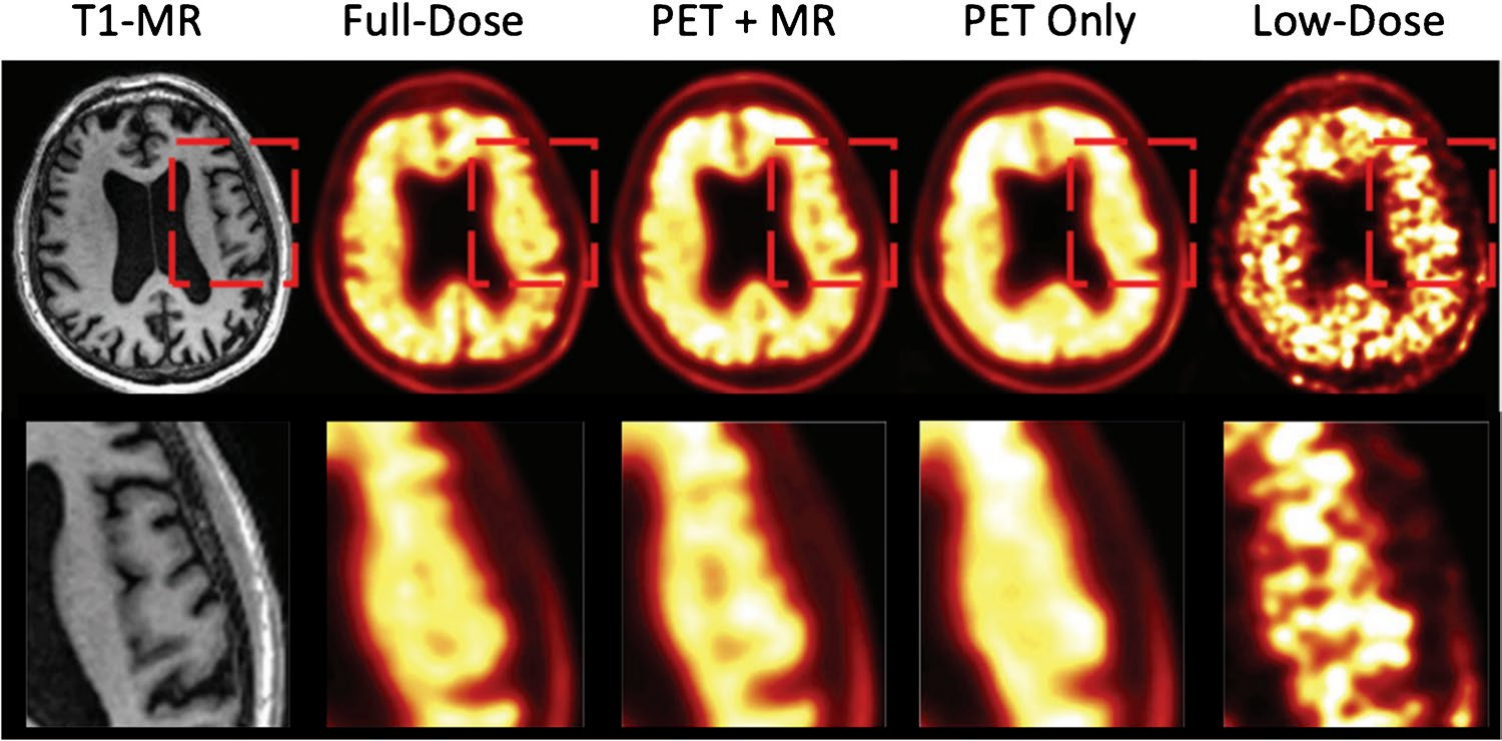
Demonstration of low-dose to full-dose mapping and the benefits of including multi-contrast MRI (PET + MR) as an input to the deep learning-based algorithm as compared to using only PET inputs (PET Only) (Images from [75])

Early work by Xiang et al. [73] showed good performance with mapping low-dose PET images with a coregistered *T*_1_-weighted MR image to diagnostic quality PET images with a dose reduction factor of 4 × using a 2D patch-based CNN. Their methods showed improved NMSE and PSNR and demonstrated the feasibility of relatively small patch-based networks. Chen et al. [75] used a residual Unet with a 2D input consisting of multi-contrast *T*_1_, *T*_2_, FLAIR and a 100-fold low-dose PET dataset for mapping to full-dose ^18^*F-*Florbetaben images. The predicted full-dose images produced amyloid positive–negative diagnostic accuracy comparable with the actual full-dose acquisition. As shown in Fig. 5, a comparison between models trained separately with PET data and multimodal PET/MR data showed that the amyloid status, PSNR, SSIM, RMSE and regional SUV accuracy improved considerably when using multimodal input because of the superior anatomical contrast in MRI.

Several groups have further investigated other model architectures and the inclusion of additional MR contrasts. Liu et al. [83] modified a Unet to include feature extractors for low-dose PET and *T*_1_-weighted MR inputs prior to concatenation of input data. Costa-Luis et al. [84] used a fully 3D input dataset with differing noise levels for full global contextual information and improved robustness to variations in noise compared to early works. Ladefoged et al. [76] investigated low-dose cardiac imaging, mapping low-dose ^18^*F*-FDG cardiac images and CT to full-dose images with a dose reduction factor of ×100  using a 3D Unet and the Huber loss function. The team further evaluated the accuracy of their method for evaluating the left ventricular ejection fraction, end diastolic volume and end systolic volume and inter-subject variation. Wang et al. [77] used water and fat MR images as additional inputs to generate full-dose PET images with a spatially weighted loss function and demonstrated reduction in SUV_mean_ and SUV_max_ quantification errors. Transferred learning was investigated in [97] as an alternative to transferring data between sites with different acquisition protocols. The results demonstrated sharing neural network parameters rather than data can produce better results for given site after fine tuning with site-specific data. An effective means of incorporating MR information into the unsupervised DIP framework was investigated by Onishi et al. [109] by extracting deep features from simultaneously acquired MR images and incorporating them into the feature space of the DIP. Including relevant MR contrast images into deep learning-based image processing has shown to improve performance over PET-only implementations.

The applicability of Poisson resampled data to accurately replicate true low-dose images was investigated by Chen et al. who used a two-stage training processes in [96], in which a residual Unet model was first trained with low-dose PET-MR images generated from full-dose images and then fine-tuned using true low-dose PET-MR images acquired from patients administered approximately 6*.*6 MBq of ^18^*F*-Florbetaben. The results were promising and demonstrated that simulating low-dose PET images by resampling can provide adequate training for optimising deep learning models. However, further validation work is still required to test the applicability of this approach under various conditions.

Sudarshan et al. [102] trained a modified Unet to map × 180 low-dose ^18^F-FDG PET brain images with coregistered *T*_1_ and *T*_2_ MRI to full-dose PET images and uncertainty maps using an uncertainty aware loss functions in both image space and sinogram space. Training the uncertainty estimator using a Bayesian framework did not require ground truth uncertainty maps. The proposed method was evaluated on varying levels of radioactivity counts, using ^18^F-FDG brain data from the ADNI database [110] for external evaluation and showed robust performance in the presence of motion artefacts. The further development of uncertainty estimation methods and their application to other imaging situations is likely to be of interest in future for developing generalisable deep learning methods with the ability to estimate the networks performance at inference.

### Perceptual and adversarial loss

Generative adversarial networks [111] are powerful in learning the underlying distribution of datasets and can improve performance over neural networks trained with commonly used analytical loss functions such as the L1 and L2 norm. Similarly, incorporating perceptual loss into neural network training using a pretrained feature extractor can help improve the perceptual quality of output images.

Kaplan et al. [90] trained 2D patch-based GANs to map ×100 low-dose ^18^F-FDG images of different anatomical regions to full-dose images, demonstrating promising proof-of-concept preliminary results. Similarly, Wang et al. [92, 93] trained a 3D patch-based GAN to map low-dose ^18^*F*-FDG PET brain images to their corresponding full-dose images. The results demonstrated that the 3D GAN outperformed both 3D Unet and 2D GAN implementations in PSNR, NMSE and hippocampal SUV bias [92] with subsequent work incorporating *T*_1_-weighted and diffusion-weighted MR images as multi-contrast input [93]. Separately, Xue et al. [87] used a patch-based mapping from low-count to standard-count whole body PET images, which improved performance relative to their experimental results from a 3D CNN. In [94], a comprehensive ablation study was performed to compare network architectures, generator initialisations and loss functions and demonstrated superior performance of a GAN that was initialised with weights trained to minimise MSE, followed by training with perceptual, adversarial and MSE loss. The cycle consistent GAN has also been applied to low-dose PET image post-processing. In [88], Lei and colleagues implemented a 3D patch-based cycle GAN to map low-dose whole body PET images to full-dose images which demonstrated superior performance in NMSE, PSNR and SUV bias compared to a standard GAN and a Unet. Subsequent work utilised a cycle GAN with the Wasserstein distance term in the loss function to perform low-dose to full-dose mapping of ^18^*F*-FDG brain images with simulated lesions [89] and whole body ^18^*F*-FDG images [85]. The results showed the cycle GAN reduced SUV bias relative to a residual Unet and conditional GAN, and improved image sharpness compared to the residual Unet. These works [85, 87–90, 92–94] collectively demonstrate the ability for adversarial loss to outperform pre-defined loss functions in specific circumstances; however, further investigations into the generalisability beyond small cohort studies will determine their clinical feasibility. More clinically relevant image quality metrics were considered in the studies by Lu et al. [95] and Jeong et al. [86]. The quantitative accuracy of ^18^*F*-FDG lesion uptake after deep learning-based low-dose processing with various neural network architectures was investigated in [95]. The findings demonstrated bias in SUV_max_ and SUV_mean_ in ^18^*F*-FDG lesions for a convolutional auto-encoder, Unet and GAN architectures with bias minimised for fully 3D networks. Jeong et al. [86] trained a GAN to map 2-min ^18^*F*-Florbetaben brain scans to corresponding 20-min acquisitions and showed the synthesised full duration images maintained diagnostic accuracy comparable to the ground truth. Future studies which include clinically relevant metrics such as diagnostic accuracy and SUV bias will become more important in demonstrating clinical feasibility of deep learning-based methods.

**Fig. 6:**
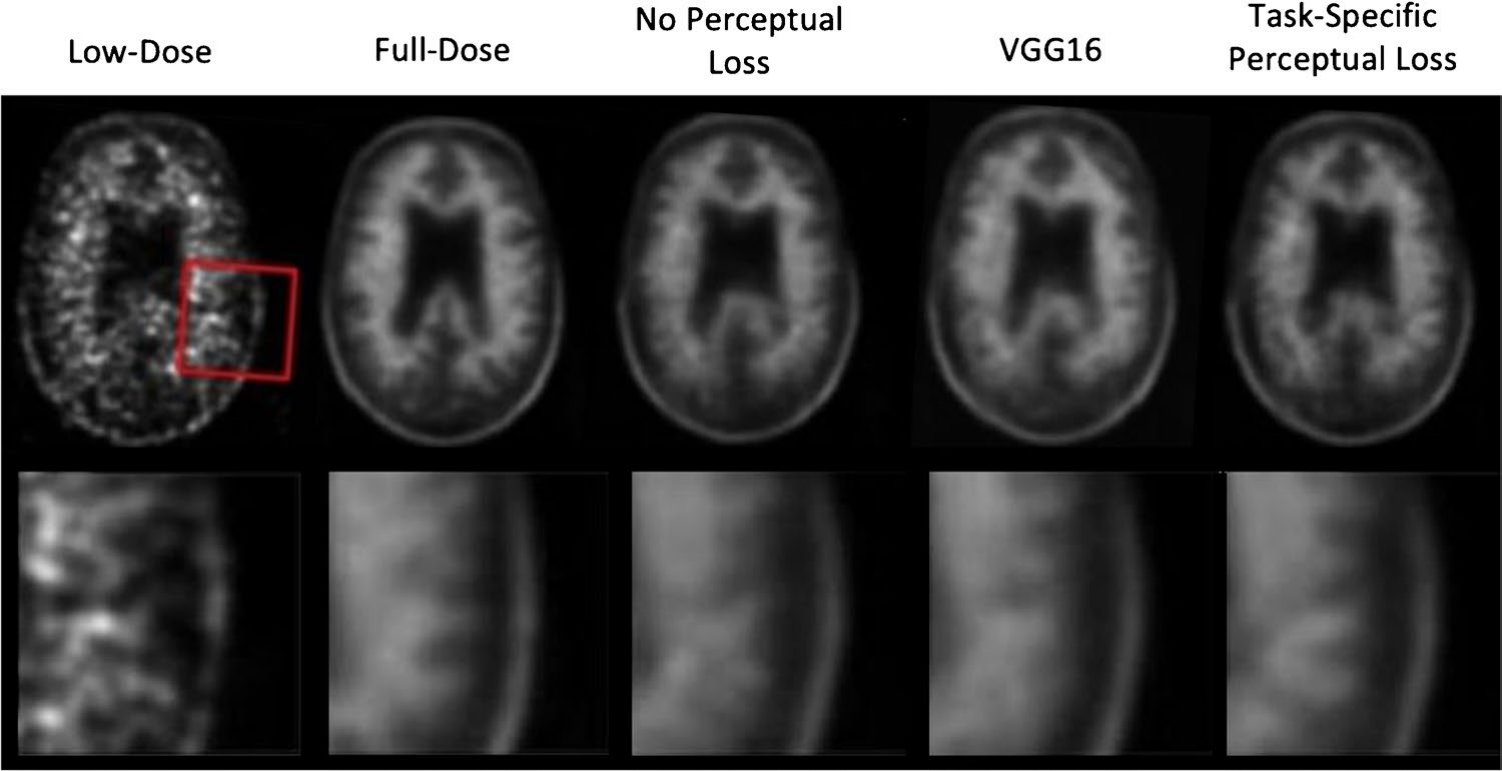
Including task-specific perceptual loss in the form of a pre-trained amyloid classifier improves diagnostic quality of synthesised full-dose images (images from [91])

Perceptual loss utilises a pre-trained feature extractor to require consistency between network outputs and ground-truth images in the learned feature space. In [99], Gong et al. used a VGG19 network architecture pre-trained with the ImageNet database of natural images as a feature extractor for perceptual loss training of a residual Unet. In a similar direction, Ouyang et al. [91] used a 2.5D GAN to map low-dose PET images to standard-dose PET trained with task-specific perceptual loss using a feature extractor pre-trained to evaluate amyloid positive or negative status. The authors demonstrated in an amyloid PET study that the feature extractor-based perceptual loss can produce diagnostic results that are comparable with multimodality MR images as input [75] (Fig. 6). It is likely that task-specific perceptual loss such as that utilised in [91] may be extended to other tracers and clinical outcomes to help preserve image features crucial for correct diagnosis.

## Review of deep learning-based resolution enhancement

The physical principles and engineering of PET systems limits their resolution. Spatial resolution is degraded primarily by the accuracy with which gamma ray interactions can be localised in the detector system as well as positron range effects which vary based on the characteristics of the radioisotope used. Temporal resolution is limited in practice by the low signal-to-noise ratio in short acquisitions due to limitations of system sensitivity and the low levels of radioactivity that can be safely administered to patients. Deep learning-based resolution enhancement in PET therefore presents a challenging task as standard supervised learning methods are often less feasible and rigorous evaluation of developed methods is limited.

### Temporal resolution

The biodistribution of a PET tracer varies temporally, with the acquired PET image indicative of the time integrated activity distribution. Investigations which consider the temporal change in tracer distribution are referred to as dynamic PET or functional PET [43] and involve temporal binning of the measured spatiotemporal signal into serial acquisitions that are reconstructed independently. Deep learning approaches to enhancing dynamic PET can be implemented either (i) in the spatial domain in which each frame is reconstructed and processed independent of the other frames, (ii) in the temporal domain in which each pixel is considered as a one-dimensional time series or (iii) spatiotemporally by the combination of both temporal and spatial information.

For spatial domain processing, the deep image prior formulation was implemented by Hashimoto et al. [112] to denoise temporally binned PET brain images using the summed image as the network input. The results showed good ability to replicate the temporal characteristics of simulated data. A spatiotemporal deep image prior which consisted of a modified Unet which simultaneously denoised all the temporal frames with a shared encoder and frame-specific decoders was then used by Hashimoto et al. [113]. Results indicated superior performance compared to Gaussian smoothing, image-guided filtering and the 3D deep image prior [112]. Adding denoising regularisation to the standard DIP formulation was also investigated [114]. Other variations on spatial domain processing techniques include work from He et al. [115] that trained a neural network to map dynamic PET and MR inputs to a downsampled composite of all frames with edge preserving regularisation and a combination of *L*_1_ and *L*_2_ loss. Finally, Cui et al. [116] used a patch-based fully connected encoder-decoder trained with simulated data to map noisy dynamic images to fully sampled dynamic images, with each temporal frame defined by a Gaussian weighted sum over all frames. The results showed improved denoising properties compared to standard MLEM reconstruction and MLEM reconstruction with TV regularisation. Processing dynamic PET data in the spatial domain allows each time point measurement to remain independent from one another which may help to prevent high-frequency temporal signal from being indiscriminately attenuated with noise, at the expense of neglecting information provided by the temporal domain. Additionally, simultaneously acquired MR contrasts are likely to be of more use when incorporated into spatial domain processing.

An alternative approach to self-supervised learning in image space is pixel-wise dictionary matching. In this approach, noisy voxel-wise time-activity curves are compared to a comprehensive library of analytical functions with specified biokinetic parameters to find the best fitting time-activity curve. The time-consuming nature of the dictionary matching process motivated the development of deep learning-based methods which encode a comprehensive simulated dictionary into a neural network [117–119]. This approach was taken by Klyuzhin et al. [120], where a library of simulated time activity curves was used to train a feed forward multi-layer perceptron for voxel-wise denoising of time activity curves. Follow-up work [121] used a patient-specific neural network trained with a simulated dictionary using parameters in the neighbourhood of parameter estimates derived from the first pass temporal data. Similarly, Wang et al. [122] used a neural network trained with simulated data to directly estimate biokinetic parameters from voxel-wise time-activity curves. Finally, Angelis et al. [123] incorporated stimulus-induced neural activations using the neurotransmitter PET model by Morris et al. [124] into a simulated dictionary and evaluated the ability of a neural network to reproduce the activation signals. The approaches taken by [120–122] produce denoised dynamic PET images which may improve the quality of dynamic information available for clinical decision-making and the approach taken in [123] extends this to include dynamic changes in tracer kinetics due to an external stimulus. However, considerations must also be made to ensure the simulated dictionary of time-activity curves includes all possibilities which may occur in practice. Implementing deep learning methods trained on simulated data may limit the utility of PET for measuring phenomena beyond the pre-determined bounds of the simulated data.

### Spatial resolution

**Fig. 7:**
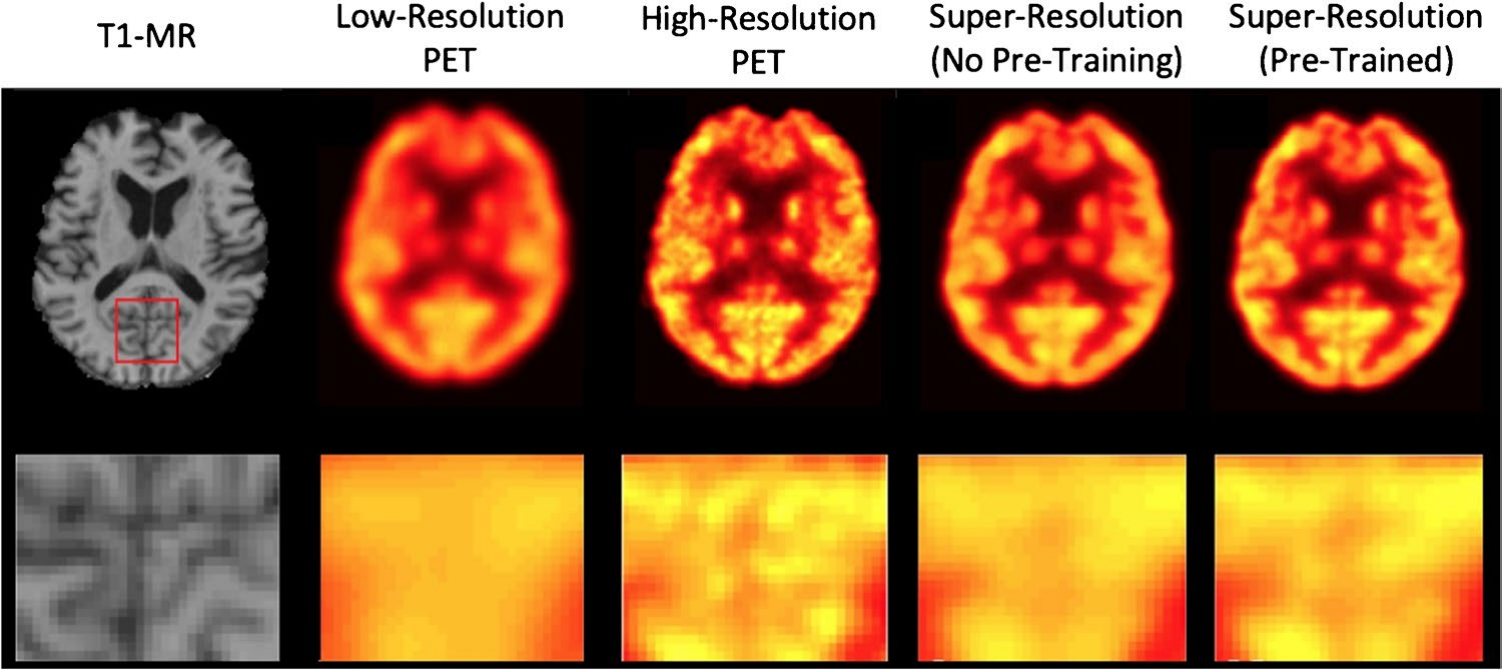
Deep learning-based spatial resolution enhancement using unpaired training with high-resolution images acquired on a dedicated brain scanner as targets. Optimal results were obtained from pre-training with synthetic data (images from [128])

While deep learning models are state-of-the-art for super resolution in computer vision and MRI [3, 125, 126], the system limitations of PET make supervised training approaches difficult due to the lack of high-resolution training data. Hong et al. [127] used digital phantoms and Monte Carlo simulations of PET systems with various crystal sizes to generate supervised learning datasets with super resolution performance evaluated in both sinogram space and image space. In [128, 129], Song and colleagues compared the performance of shallow and deep network architectures for PET super-resolution. They used images acquired from high-resolution dedicated brain imaging systems as a supervised training target with post smoothing applied to simulate low-resolution scans. Further work incorporated an initial super resolution network trained on synthetic data into a GAN to map standard resolution PET images and multi-contrast MR images to high-resolution images. The method used unpaired high-resolution PET brains acquired on a dedicated brain PET scanner as ground truth (Fig. 7). Methods which use deep neural networks to parameterise computationally expensive analytical resolution enhancement techniques have been developed including performing point-spread function-deconvolution operation as post-processing using a Unet [130] and also suppressing Gibbs artefacts generated from point-spread function modelling [131]. Similarly, Schramm et al. [132] used a CNN to map *T*_1_-weighted MR images and OSEM reconstructed PET brain images to MR-guided OSEM reconstructions which use the asymmetric Bowsher prior [28] as regularisation. The inability to generate high-resolution ground-truth images limits the application of deep learning-based super-resolution; however, the methods of unpaired training [128] and simulating high-resolution data [127] may provide feasible alternatives to supervised training. Evaluating the performance of such techniques in the absence of ground truth data presents an additional challenge.

## Discussion

The last 5 years have shown artificial intelligence and deep learning models to be highly versatile tools for application to PET image processing. Many techniques developed in computer vision have been successfully applied to CT and MR imaging and translated to PET. While most of the results to date have been obtained using small cohort studies with relatively narrow demographics, studies which focus on reliable clinical implementation will become of more relevance in the near future. However, the ultimate impact that deep learning models will have on PET image reconstruction and post-processing in the future will depend on whether several key obstacles can be successfully addressed.

The generalisability of a neural network to data outside of the training set, referred to as out of distribution (OOD) data, is a critical limiting factor for deep learning-based image processing. In the context of diagnostic imaging, OOD has the potential to produce false readings with potentially dire consequences for patient outcome. Furthermore, as neural networks are often poorly conditioned [133], it is challenging to define the limits of their applicability with confidence. Consequently, the validity in extrapolating the performance of neural networks from small cohort studies to the clinical setting is currently uncertain. This is especially the case for application to broad patient diagnostic categories with variable pathological characteristics. Furthermore, the temporal variation in imaging systems may progressively degrade the performance of a neural network in an unknown manner. Routine clinical implementation of deep learning methods requires the image enhancement algorithm to be demonstrably robust to OOD data to provide clinicians with confidence when making diagnostic decisions using the enhanced PET images. New methods are needed to characterise, validate, and improve the application of neural network models to out of distribution data. A comprehensive standard for the evaluation of deep learning-based PET image processing algorithms is needed to provide a quantitative and comparable evaluation of the performance of models. The standard should include an evaluation of the model generalisability under varying conditions, as was shown in some of the studies reviewed in this work [89, 102].

Novel methods to improve the generalisability of deep learning models will help facilitate their clinical use. The task-specific nature of deep learning-based methods typically limits their applicability to specific tracers and anatomical regions. Physically justifiable regularisation terms in training, derived from the nature of radiation transport and incorporated into a model of the PET system, may help to ensure physically consistent results for OOD data, without the need for prior assumptions regarding the physiology of a tracer. This approach could lead to the development of deep learning algorithms which generalise beyond a single tracer or anatomical region that would make training and implementation more logistically feasible. Uncertainty estimation methods [134, 135] can quantify the quality of predictions for OOD data. Integration of these approaches in deep learning-enhanced PET image reconstruction and post-processing will be crucial to provide quantitative metrics to assist physicians in their assessment of the diagnostic confidence of features in PET images. Future studies are required to investigate the clinical utility of uncertainty maps for accurately characterising anomalous outputs from neural networks. Studies of this nature would help boost diagnostic confidence in the clinical setting.

The availability of high-quality training data is a major limitation in the development of deep learning-based methods. Supervised learning of deep neural networks requires high-quality training data to comprehensively describe the variations in the image features for images drawn from the population to which the network will be applied. In practice, there are several logistical and ethical issues associated with accessing the necessary imaging data and related information. The sensitive nature of medical information poses a problem for collecting comprehensive datasets from many sources for research and commercial development purposes. Anonymised open access medical imaging datasets can help make research feasible for groups without the capabilities to produce data. Researchers are encouraged to make new datasets open access when possible. The risks of shared DICOM metadata being incorrectly anonymised are manageable; however, providing adequate protection against facial recognition software [136] is more complex and may involve altering the image data itself. Methods such as federated learning [137] can provide a more robust means for anonymous use of medical data without the need for explicit data transfer between parties. Federated learning approaches are likely to become essential for large-scale validation and commercial development of deep learning-based medical imaging software.

The issue of adequate data availability is more fundamental for applications such as resolution enhancement where the production of high-quality ground truth data is limited by the PET system performance and radiation risks. Unsupervised learning methods may provide a means to utilise deep learning models in such applications. Alternatively, supervised learning with synthetic data may prove useful, with Monte Carlo-based methods capable of accurately modelling the PET acquisition process including physical effects such as scatter and attenuation. Computational power is usually a limiting factor for generating volumetric PET images with Monte Carlo modelling. However, recent work in this field has demonstrated significant gains in computational efficiency [138, 139] that make it feasible to generate a volumetric PET image in tens of hours. While this may prove useful in generating otherwise unattainable ground truth data, its utility will depend fundamentally on the ability to accurately model physically realistic data. It is the case that using simulated data may compromise the utility of PET as an investigative tool for measuring phenomena beyond the scope of a pre-defined library of simulated data.

Additional studies to evaluate the performance of deep learning-based methods across multiple sites will be crucial to demonstrate the performance of commercially viable deep learning-based methods. While those works which evaluated performance across sites [100, 101] showed promising results, large-scale validation on a range of tracers and anatomical regions will be required to identify the shortcomings of deep learning-based methods in a clinical setting. Such studies will inform the development of commercially available deep learning-based software which should perform consistently across sites. Future studies should also focus on evaluating clinically relevant metrics in addition to quantitative image quality measures to identify the net benefits of clinical implementation.

## Conclusion

Positron emission tomography offers a means of measuring physiological processes in vivo and plays an essential and unique role in clinical patient care and scientific research. This review provides an overview of the current state-of-the-art deep learning methods and future research directions in image reconstruction and post-processing for PET image enhancement. The integration of newly developed methods from the field of artificial intelligence into conventional PET image processing will further enhance the breadth of capabilities of PET imaging. Deep learning can be incorporated into image reconstruction as a purely data-driven mapping from raw data to images, or as a regularisation term in combination with conventional data consistency. Post-processing techniques offer a multitude of practical ways to integrate deep learning into image processing frameworks.

The ultimate impact of deep learning models on PET image reconstruction and post-processing will depend on whether several key obstacles can be successfully addressed. The generalisability of a neural network to out of distribution data is a critical limiting factor for deep learning-based image processing. Emerging uncertainty estimation methods have the potential to quantify the quality of predictions for OOD data. The lack of high-quality training data is a further major limitation for the development of deep learning-based methods with supervised learning. Unsupervised learning techniques and high-quality synthetic data may help to mitigate this issue. Federated learning offers a means of utilising data across multiple sites without explicit transfer of medical images and the associated risk of loss of patient confidentiality. Although several key challenges exist, it is apparent that deep learning will play a pivotal role in the future of PET imaging.

## Data Availability

No original data or materials were used in this work.
